# Distribution of Lipids and Prevalence of Dyslipidemia among Indian Expatriates in Qatar

**DOI:** 10.1155/2021/8866784

**Published:** 2021-03-05

**Authors:** R. Nirwan, D. Singh

**Affiliations:** ^1^Specialist Laboratory Medicine/Hematologist Aster DM Healthcare, Qatar; ^2^Cardiff Medical Centre and Skin Cancer Clinic, Cardiff, NSW 2285, Australia

## Abstract

**Background:**

Dyslipidemia is a significant risk factor for cardiovascular diseases (CVD). If detected and managed in the early stages of life, can reduce morbidity and mortality associated with CVD in a vulnerable population. Out of the 94 expatriate nationalities in Qatar, Indians constitute the most prominent single nationality, accounting for 21.8% of the total population (2,773,885 in 2019). This study aims to determine the status of the lipid profile among Indians in Qatar. *Study Design*. We conducted an observational retrospective study on lipid profile test data of Indian expatriates visiting a private healthcare facility in Qatar from Oct 17 to Oct 2018 to evaluate the gender and age-specific distribution of lipids and the prevalence of dyslipidemia.

**Results:**

Among the total 4483 Indian expatriates (3891 men and 592 women), the mean (SD) mg/dL levels of total cholesterol (TC), triglycerides (TG), and low-density lipoprotein cholesterol (LDL-C) were higher in men TC 196.9 (40.6), TG 168.9 (114.6), and LDL-C 122.9 (37.2) mg/dL compared to women TC 185 (38.1), TG 117.7 (78.2), and LDL-C 114.1 (31.1) mg/dL, *p* value < 0.0001. Utilizing predefined National Cholesterol Education Program-Adult Treatment Panel III (NCEP ATP III) limits to categorize dyslipidemias; the greater prevalence of elevated TC, TG, and LDL-C was noted in men 44.7%, 45.8%, and 40.9% than women 31.6%, 22%, and 28.7%, respectively. However, women had higher levels of mean high-density lipoprotein cholesterol (HDL-C) as 47.1 (9.8) mg/dL vs. 40.6 (8.3) mg/dL in men, *p* value < 0.05, the prevalence of dyslipidemia, low HDL-C was also more 65.7% vs. 48.9% in women than men. With age, men showed a declining trend while women showed a rising trend for mean lipid levels as well as for the prevalence of dyslipidemia, high TC, TG, and LDL-C (*p* value < 0.0001). The mean HDL-C cholesterol increased, and the prevalence of dyslipidemia, low HDL-C decreased with age in both the genders.

**Conclusion:**

Our results demonstrate the higher mean lipid levels and prevalence of atherogenic dyslipidemia among Indian expatriate men than women counterparts at the younger age group. The screening programs and awareness campaigns must be initiated to prevent the early onset of dyslipidemia induced atherosclerosis leading to CVD. Future controlled studies are needed to estimate the prevalence of dyslipidemias among Indian migrants in Qatar.

## 1. Introduction: A Global Concern

The increase in urban spread and influence of industrialization in the developing countries increased the burden of chronic noncommunicable diseases (CNCD) like cardiovascular diseases [1]. Cardiovascular diseases took 17.6 million out of 40.5 million deaths in 2016 globally. The WHO has reported ischemic heart disease and stroke as the top causes of mortality [2]. In Asia and the Middle East, CVDs are among the most prevalent and debilitating diseases. There is a well-established association between abnormal lipid concentrations and the etiogenesis of atherosclerosis, a modifiable risk factor for CVD [3]. Epidemiologic data on incidence and determinants of dyslipidemia need to be updated in most nations to assess the risk of CVD, though not expressed but initiated at a younger age.

Early identification by screening can prevent the onset and progression of atherosclerosis.

This study proposes to document the distribution of lipids and the prevalence of dyslipidemia among the Indian expatriate population in Qatar. What further intrigued us was if there were any changes in the pattern of dyslipidemia by age and gender.

Qatar is a rapidly developing, wealthy sovereign country, an ethnically and culturally diverse region having expatriate populations from different parts of the world. Out of 2.7 million population (76% men and 24% women), 60% are South Asians with 21.8% Indians [4]. Dyslipidemia includes abnormal levels of lipid-elevated levels of total cholesterol (TC), low-density lipoproteins (LDL-C), nonhigh-density lipoprotein (NHDL-C), triglycerides (TG), and low levels of high-density lipoproteins (HDL-C). These plasma lipid disorders may be primary, occurring due to the interaction of genetic susceptibility and environmental risk factors or secondary, occurring as a result of other disorders (e.g., diabetes, hypothyroidism, and nephrotic syndrome). Due to the paucity of local data in the Asian and Middle East regions, physicians often must refer to major international guidelines [5]. Therefore, most of the laboratories use NCEP ATP III (2001) criteria for the screening and management of dyslipidemia [6]. LDL-C has long been found as the primary target of cholesterol-lowering therapy. If TG is more than 200 mg/dL, lowering of non-HDL-C-C is considered a secondary goal, especially for obese, and those were having metabolic syndrome. Management of TG is on a priority if more than 500 mg/dL, to reduce the risk of pancreatitis. Recently, 2018 AHA/ACC Guidelines have considered South Asian ancestry and postmenopausal women as risk enhancing factors for CVD [7]. Lipid Association of India has interpreted the newer guidelines with the Indian perspective giving more emphasis on triglyceride and HDL-C axis management due to specific genetic influences on lipid levels and structures [8].

## 2. Methodology

### 2.1. Data Collection

We performed a retrospective observational study on lipid profile test data requested from Oct 2017 to Oct 2018, at four branches of Aster DM Healthcare, Qatar, a private healthcare provider: Dr. Moopen's Aster Hospital, Doha; Aster Medical Centre Plus, Al Muntazah; WellCare Polyclinic, Al Rayyan; and Aster Medical Centre, Al Khor coinciding with four major populous constituencies in Qatar [9].

Total cholesterol, triglycerides, and HDL-C cholesterol were measured by enzymatic methods, and LDL-C cholesterol was calculated using the Freidwald equation LDL − C Cholesterol(mg/dL) = Total Cholesterol–(HDL − C + Triglycerides/5). We processed the fasting samples on fully automatic Biochemistry Analyzers using high-end quality reagent kits following strict adherence to quality control protocol; CV% for internal quality control and SDI, % deviation, and TS for external quality control from Randox in our all laboratories.

### 2.2. Reference Values

We used National Cholesterol Education Program-Adult Treatment Panel III (NCEP ATP III) limits to define dyslipidemia for cholesterols and triglycerides [6]. Borderline high levels are defined as TC >200 mg/dL, LDL-C >130 mg/dL, TG >150 mg/dL, and low HDL-C <40 mg/dL in men and <50 mg/dL in women. Very high levels are defined as TC >240 mg/dL, TG >500 mg/dL, LDL-C >170 mg/dL, and combined atherogenic dyslipidemia as high TG and low HDL-C.

### 2.3. Statistical Analysis

We exported the database from electronic media to Microsoft Excel Windows 10 for descriptive statistical analysis. Central tendencies for continuous variables were expressed as means and standard deviation and as percentages for categorical variables. The correlation was done using Student *t*-test or Anova for means and chi-square for proportion through the SPSS 16.0 statistical software package (SPSS, Inc., Chicago, IL), and considered *p* value < 0.05 as statistically significant [10].

## 3. Results

### 3.1. Population Characteristics

After excluding other nationalities and incomplete entries for age and other data from 6098 requested lipid profile tests, we included data of 4483 Indian expatriates, 3891 men, and 592 women aged 20-69 years. The mean (SD) age was 39 ± 8.9 years. The highest number of subjects, 44.2%, was in the 30-39 age group, followed by 29.3% in 40-49 years. According to statistics of Qatar in 2018, 74% are men, and 26% are women with 73% population in 25-64 years and only 1% above 64 years. The sample size was calculated according to population statistics in Oct 2018 [11].

### 3.2. Lipid Distribution by Gender

The mean (SD) mg/dL levels of various lipids in the entire study populace were as follows: TC 195.4 (40.5), TG 162.2 (111.8), LDL-C 121.8 (36.5), and HDL-C 41.5 (8.8) as shown in Table 1. Men had higher mean (SD) levels for TC 196.9 (40.6), TG 168.9 (114.6), and LDL-C 122.9 (37.2) mg/dL than women TC 185 (38.1), TG 117.7 (78.2), and LDL-C 114.1 (31.1) mg/dL, *p* < 0.0001, while the HDL-C was higher in women 47.1 (9.8) mg/dL vs. 40.6 mg/dL in men, *p* < 0.0001. As we can notice, the mean TC and LDL-C are below, and the mean TG levels are higher than the cut-off values for dyslipidemia definition. Mean HDL-C levels are also lower than cut off.

**Figure 1:**
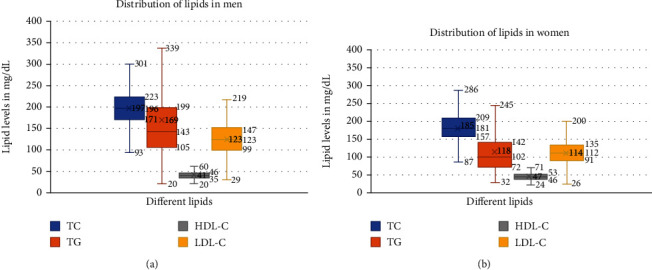
Distribution of lipids among men and women. (a, b) Distribution of lipids among men and women (box and whisker plot). The upper whiskers for TC, TG, and LDL-C are more in men than women, while high for HDL-C in women. TC has the highest level among all lipids in men with a median of 196 mg/dL, mean 197 mg/dL, and interquartile ratio (IQR) is 52 (223-171). The box of TG shows the median of 143 mg/dL, mean 168.9 mg/dl, and IQR is 94 (199-105) in men.

Box and whisker graph (Figures 1(a) and 1(b)) displays the distribution of lipids [10]. The interquartile ratio (IQR), the difference between the 25th and 75th percentile, is almost similar in men/women for TC (52/52), LDL-C (48/44), and HDL-C (11/7), respectively. For HDL-C, the upper whisker, i.e., 4th quartile, is at 60 mg/dL in men and 71 mg/dL in women. The IQR for TG was more in men (94) than women (70) as clear from the more height of the box. The long whiskers also denote the wider range of triglycerides in men 20-339 mg/dL than in women 32-245 mg/dL. The median for TG was 143 mg/dL in men and 102 mg/dL in women. We observed TG >500 mg/dL in 54 men in our study, while only two women had TG >500 mg/dL.

### 3.3. Lipid Distribution by Age

We performed ANOVA multiple comparisons in total population and analyzed the difference between overall mean levels in different age groups. The statistical analysis showed that age groups 30-39 and 40-49 presented higher mean levels of TC and TG (30-39: *p* < 0.0001, 40-49: *p* < 0.001), compared to the other age groups. Age groups 30-39, 40-49, and 50-59 showed higher mean levels of LDL-C (30-39 and 40-49 age groups: *p* < 0.0001, 50-59: *p* < 0.0001) than 20-29 age-group, which presented the lower mean LDL-C concentration (107.3 ± 27.55 mg/dl). Regarding HDL-C, age group 30-39 presented the lower mean levels (40.68 ± 8.55 mg/dl, *p* < 0.0001) than the 20-29 age group.

As shown in Table 1, mean lipid levels in different age groups among men, 20-29 years age group had lower values for all four lipids than 30-39 years age group. The highest values were in 30-39 years for TC 199.8 (40.8), TG 179.1 (128.9), and LDL-C 124.7 (37.4) mg/dL and lowest in 60-69 years age group TC 180.7 (180.7), TG 133.9 (80.3), and LDL-C 111 (37.3) mg/dL, exhibiting a declining trend with age, *p* value < 0.0001. In contrast, the pattern was rising for mean HDL-C levels lowest 39.8 (8) mg/dL in 30-39 years and highest 43.7 (9.7) mg/dL in 60-69 years, *p* value < 0.0001.

On the other hand, women showed a different pattern, lowest values of the mean (mg/dL) TC172.4 (29.1), TG 98.3 (52.9), and LDL-C 106 (26.1) in 20-29 years, and highest at TC 203.3 (51.6), TG 156.9 (125.6), and LDL-C 122.2 (36.2) in 50-59 years age group. Noted a decline after 60 years for these lipids. However, the mean HDL-C level was higher in women than men at all age group intervals; we noted a declining trend in both genders with age.

### 3.4. Prevalence of Dyslipidemia

**Figure 2:**
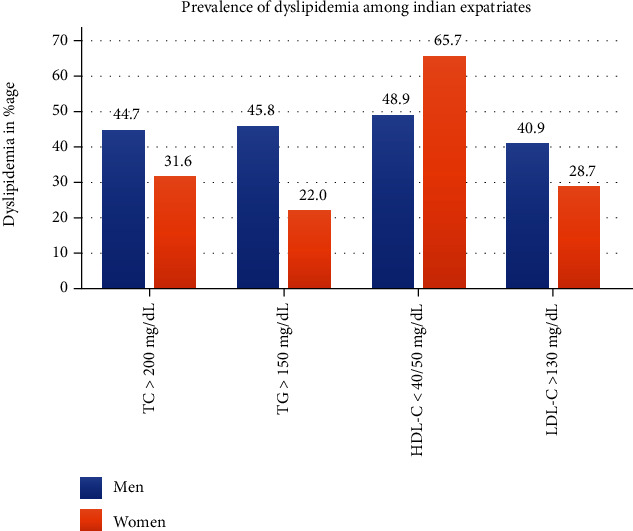
Prevalence of dyslipidemia. Prevalence of various dyslipidemias among Indian expatriates in Qatar: prevalence of dyslipidemia for total cholesterol, triglycerides, and low-density lipoprotein cholesterol is higher in men, while the prevalence for high-density lipoprotein cholesterol dyslipidemia is significantly higher in women (*p* value 0.0001).

According to the National Cholesterol Education Program-Adult Treatment Panel III (NCEP ATP III) criteria for categorization of dyslipidemias, the highest prevalence was noted for dyslipidemia, low HDL-C 51.1% followed by elevated TC 43%, TG 42.7%, and LDL-C 39.3% in total subjects. Men had a high prevalence of dyslipidemia as compared to women counterparts high TC 44.7%, vs. 31.6%; high TG-45.8%, vs. 22%; high LDL-C-C 40.9%, vs. 28.7%, respectively, *p* value < 0.0001 (Figure 2: histogram).

The prevalence of dyslipidemia, low HDL-C was higher in women 65.7% than men 48.9% *p* values < 0.0001. As the cut-off value for dyslipidemia, HDL-C is <50 mg/dL, in women despite higher mean HDL-C levels, the dyslipidemia prevalence was also high.

The prevalence of atherogenic dyslipidemia; high TG and low HDL-C in the study population was observed in 24.3% of subjects (26.9% men and 17.4% women). The overall prevalence of dyslipidemia in the total Indian expatriate populace was 80% having at least one abnormal lipid level, and 7% had dyslipidemia for all four lipids.

According to ATP III criteria, very high TC >240 mg/dL was seen in 13.1% and TG >500 mg/dL in 1.2% (1.4% men and 0.34% women).

### 3.5. Prevalence of Dyslipidemia by Age

**Figure 3:**
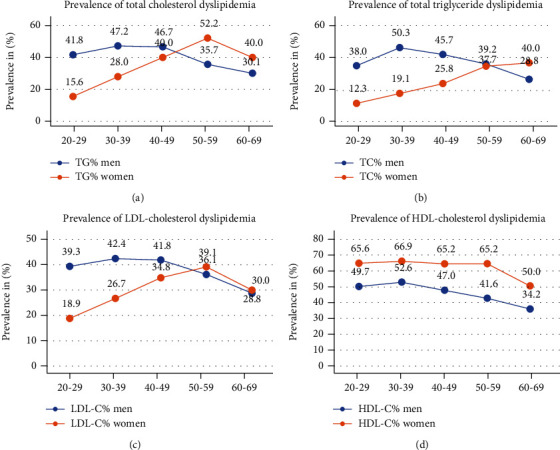
Age group-specific trend of prevalence of dyslipidemia among Indian expatriate men and women. (a–d) Comparison of prevalence of dyslipidemia for various lipids in different age groups among men and women. Prevalence of dyslipidemia for total cholesterol, triglycerides, and LDL-C cholesterol is higher in men at younger age groups than women, *p* value < 0.0001. The mean TC, TG, and LDL-C in women showed a modest rise and supersede the levels in men at 50-59 years age group. After 60 years, the mean levels are decreasing in both genders except TG. The prevalence of HDL-C dyslipidemia is higher in women at all age groups, and a declining trend is noted in both genders (*p* value < 0.0001).

As seen in Table 2 and Figure 3, the prevalence of dyslipidemia, high TC, TG, and LDL-C with age in men, low values at 20-29 years, peak at 30-39 years, and after that presented a decline, reaching to minimum values at 60-69 years age group, *p* value < 0.05 and negative chi-square value. Women had minimum levels of prevalence of dyslipidemia for all four lipids at 20-30 years, an ascending slope, with a pinnacle at 50-59 years and a dip at 60-69 years. Descending patterns was appreciated for the prevalence of dyslipidemia, low HDL-C in both genders with age.

## 4. Discussion and Conclusion

Our study results revealed higher mean lipid levels among Indian expatriates in Qatar compared to reported levels in some studies for Indians living in India [12, 13]. Total cholesterol in our study was 195.4 mg/dL, and one study reported interstate differences ranging from the lowest 164 mg/dL in Andhra Pradesh to 197 mg/dL in Kerala [14].

The prevalence of overall dyslipidemia was 80%. The prevalence of dyslipidemia, high TC (43%) was more than the reported prevalence on the National health portal, 25-30% for urban and 10-20% for the rural population in India [15]. A study conducted in 2016, in South India by Krishnan et al., however, reported a higher prevalence of dyslipidemia, high TC 52.3% in Kerala than our study 40.6% [16].

In our study, expatriate Indian men had high mean TC 196.9 mg/dL than women 185 mg/dL, unlike studies conducted in India, which reported higher mean levels in women (Table 3(a)). However, the mean TG and LDL-C were greater in men than women in our study, like the studies in India [12, 13, 17, 18].

Men had a higher prevalence as well, for dyslipidemia, high TC 44.5% compared to women 31.9%, while studies in India have reported either similar or lower values in women.

Interestingly, a study in 2016 on a large database of 67000, Gupta et al. reported a higher prevalence of dyslipidemia, high TC in women (Table 3(b)) [12].

The prevalence of dyslipidemia, elevated TG was higher in men 45.7% than in women 22.1%, like other studies in India. The mean HDL-C levels were higher in women in our study, and the prevalence of dyslipidemia, low HDL-C was also higher for women 65.8% than men 47.8%, parallel to other Indian studies which reported even as high as 76% for women in the study [19].

Ours is the first study to assess gender differences in trends for the prevalence of dyslipidemia by age among Indians in Qatar. There were not many studies available for following an age-associated trend of mean and prevalence of dyslipidemia. A declining trend with age after 30-39 years in our study was similar not only to the studies in India [12], in other countries, as well such as China and Kuwait [20, 21].

According to the STEPwise survey conducted in 2012, on the Qatari population, mean TC and prevalence of dyslipidemia, elevated TC (166.9 mg/dL and 19.1%) were lower than our study population (195.3 mg/dL and 44.5%), respectively [22]. The prevalence of dyslipidemia, low HDL-C was lower 45.5%; in contrast to our study, it was more in men 51.9% than women 39.4% and reported a rising trend with age in both the genders.

NCHS data brief (2015-2016) in the United States using high TC, more than 240 as the cut off reported the peak for the prevalence of dyslipidemia, TC in the older age group 40–59 (16.5% in men 17.7% in women) than 30-39 years for Indians in our study group. Declining after age 60 and over is 6.9% in men and 17.2% in women [23].

In addition to genetic predisposition for CVD and other associated risk factors like DM and hypertension, high cholesterols among Indian men at a younger age group can be attributable to career and work-related stress, reduced physical activity, carbohydrate-rich diet intake, and tobacco use [24]. The cardioprotective hormones attribute to the low prevalence of dyslipidemia in premenopausal women [24].

Indians not only have low levels of HDL-C also have proinflammatory small-dense dysfunctional HDL-C particles [25]. A recent study in Qatar reported a higher prevalence of CVD among South Asians [26]. Few studies evaluated the CVD risk factors on South Asian migrants in Europe and the United States on Punjabis in London 1995 [27], MASALA study in 2013 [28], and on Gujratis in Britain 2006 [29]. These studies reported higher mean levels for TC and TG and low HDL-C in South Asian migrants than their native counterparts. Both vegetarian and nonvegetarian Indians share a higher prevalence of coronary artery diseases considered “Indian Paradox*”* [25, 30]. Some studies reported high mean TG and low HDL-C levels with comparable LDL-C levels as the major determinants of atherogenic dyslipidemia among South Asian migrants as compared to other ethnic groups.

Though genetic influence cannot be altered, most metabolic disorders and cardiovascular diseases are preventable by addressing behavioral risk factors such as tobacco use, unhealthy diet and obesity, physical inactivity, and harmful use of alcohol using population-wide strategies. WHO (2013) recommend the BEST BUY approach, promoting the healthy food choice at the individual level and by providing subsidiaries on fruits and vegetables, taxation on food rich in total fat, saturated fats, more excise duty on tobacco and alcoholic products, and construction of cycling tracks for promoting physical activities at the public level.

Global concerns about the prevalence of NCD have been rightly found in this study [1]. The results derived from our nationally representative sample-sized study indicate that Indian expatriate men have higher levels of atherogenic dyslipidemia at a young age compared to women counterparts. The population at higher risk needs early detection by screening and management by medicines and counseling, as appropriate. More awareness and screening programs are required to prevent the early onset of atherosclerosis due to dyslipidemia in this age group. The preventive measures are cost-effective achievable and sustainable goals than the exorbitantly expensive curative measures after the onset of a cardiac event. This study provides a baseline for future longitudinal studies to evaluate modifiable metabolic risk factors among Indian expatriates.

## 5. Strengths and Weaknesses of the Study

The large sample size and multisite data collection from populous cities of Qatar are the strengths of this study. To nullify the bias from hospital-based data collection to represent the country population in epidemiological studies, we have included outpatients and medical examination lipid profile data [31]. The weaknesses were the first being a retrospective study; we could not divide data according to different regions of India. Though we could not ascertain in our study or from any government website, this is a known fact that most Indian expatriates in Qatar are from South India. There are reports of significant interstate variations in the baseline lipid levels in India, according to the Human Development Index, more developed states have high mean TC, and less developed states have high mean TG levels [18, 32, 33]. On the second one, we did not include other details like weight, blood pressure, glucose levels, and treatment history of subjects. The third weakness was the migrant population in Qatar is either skilled or unskilled laborers, and the proportion of the women and elderly population was low in comparison to adults.

## Figures and Tables

**Table 1:** Mean serum lipids among Indian expatriates in Qatar, by gender and age.

	Number	Total cholesterol, mg/dL		Triglycerides, mg/dL		HDL-C, mg/dL		LDL-C, mg/dL	
		Mean	SD	Mean	SD	Mean	SD	Mean	SD
Grand Total	**4483**	**195.4**	**40.5**	**162.2**	**111.8**	**41.5**	**8.8**	**121.8**	**36.5**
Men	**3891**	**196.9** ^**a**^	**40.6**	**168.9** ^**b**^	**114.6**	**40.6** ^**c**^	**8.3**	**122.9** ^**d**^	**37.2**
20-29	455	192.9	39.4	154.4	92.5	40.3	8.0	121.9	35.3
30-39	1744	199.8	40.8	179.1	128.9	39.8	8.0	124.7	37.4
40-49	1157	198.7	39.9	168.5	106.0	41.2	8.5	123.9	36.9
50-59	462	188.2	40.2	151.4	96.4	42.0	8.7	116.8	37.4
60-69	73	180.7	44.6	133.9	80.3	43.7	9.7	111.0	37.3
IQR		53		94		10		48	
Women	**592**	**185.0** ^**a**^	**38.1**	**117.7** ^**b**^	**78.2**	**47.1** ^**c**^	**9.8**	**114.1** ^**d**^	**31.1**
20-29	122	172.4	29.1	98.3	52.9	46.7	9.6	106.0	26.1
30-39	236	183.2	35.4	109.2	75.4	47.1	9.8	113.7	30.1
40-49	155	190.1	37.5	127.5	65.2	46.4	9.1	118.1	31.9
50-59	69	203.3	51.6	156.9	125.6	49.4	11.4	122.2	36.2
60-69	10	179.7	43.6	135.9	49.8	50.3	11.5	103.2	42.3
Pearson coefficient		0.206		0.22		0.065		0.124	
*p* value		<0.0001		<0.0001		>0.05		<0.005	
IQR		52		70		13		44	

Comparison of gender-specific serum lipid levels: Indian men had high mean (SD) levels of TC, TG, and LDL-C cholesterol (*t*-test: a, b, and d; *p* < 0.0001) than women. Indian expatriate women had high mean HDL-C levels (c; *p* < 0.0001).

**Table 2:** Prevalence of Dyslipidemia among Indian Expatriates in Qatar, by gender and age.

	Total number	Total	Cholesterol, >200 mg/dL	Triglycerides, >150 mg/dL		HDL-C, <40/50 mg/dL		LDL-C, >130 mg/dL	
		Number	%	Number	%	Number	%	Number	%
Total	4483	1928	43.0	1912	42.7	2293	51.1	1761	39.3
Men	3891	1741	44.7	1782	45.8	1904	48.9	1591	40.9
20-29	455	190	41.8	173	38.0	226	49.7	179	39.3
30-39	1744	824	47.2	878	50.3	917	52.6	740	42.4
40-49	1157	540	46.7	529	45.7	544	47.0	484	41.8
50-59	462	165	35.7	181	39.2	192	41.6	167	36.1
60-69	73	22	30.1	21	28.8	25	34.2	21	28.8
Chi-square			29.34		42.29		27.44		11.23
*pvalue* ^∗^ *significant*			*<0.0001* ^∗^		*0.0001* ^∗^		*0.0001* ^∗^		*0.02* ^∗^
Women	592	187	31.6	130	22.0	389	65.7	170	28.7
20-29	122	19.0	15.6	15.0	12.3	80.0	65.6	23.0	18.9
30-39	236	66.0	28.0	45.0	19.1	158.0	66.9	63.0	26.7
40-49	155	62.0	40.0	40.0	25.8	101.0	65.2	54.0	34.8
50-59	69	36.0	52.2	26.0	37.7	45.0	65.2	27.0	39.1
60-69	10	4.0	40.0	4.0	40.0	5.0	50.0	3.0	30.0
Chi-square (age groups)			34.85		20.99		1.285		12.77
*p* value ^∗^significant			0.0001^∗^		0.0003^∗^		0.8639		0.01^∗^
Chi-Square (men/women)			36.29		119.4		57.87		31.92
*pvalue* ^∗^ *significant*			*<0.0001* ^∗^		*<0.0001* ^∗^		*<0.0001* ^∗^		*<0.0001* ^∗^

Age and gender-specific prevalence of dyslipidemia in the study population: Indian expatriate men and women showed a significant association of prevalence of dyslipidemias for total cholesterol, triglycerides, low-density lipoprotein cholesterol, *p* value < 0.05. Among women, the correlation with age is not significant for the prevalence of HDL-C dyslipidemias, *p* value > 0.05.

**Table:** (a) Comparison of mean lipid levels of Indian expatriates in Qatar with other studies on Indians

Men	Year	Sample size	TC mean (SD) mg/dL	HDL-C mean (SD) (mg /dL)	TG, mean (SD) mg/dL	LDL-C mean (SD) mg/dL
This study	2019	3869	197 (40.5)	41 (10.4)	166 (100.9)	123 (36.8)
R. Gupta^12^	2016	4452	164.3 (46.7)	39.7 (10.20)	146.4 (160.2)	101 (39.1)
T. Sekhri et al.^19^	2014	10642	186.1 (40.6)	42.5 (11.5)	NA	NA
S. Guptha et al.^13^	2014	3388	178.4 (39)	44.9 (11)	162.5 (83)	102.5 (33)
Women						
This study	2019	579	185 (38.4)	47 (9.5)	117 (70.8)	11431.2()
R. Gupta^12^	2016	1552	183.1 (44.1)	47.0 (12.3)	128 (92.8)	113 (37.3)
T. Sekhri et al.^19^	2014	1966	181.7 (36.6)	46.5 (11.4)	NA	NA
S. Guptha et al.^13^	2014	2735	184.6 (39)	51.1 (11)	143.7 (83)	106.2 (33)

**Table:** (b) Comparison of prevalence of dyslipidemia among Indian expatriates in Qatar with other studies on Indians

Men	Year	Sample size	TC >200 mg/dL %(N)	HDL-C <40 mg/dL % (*N*)	TG >150 mg/dL % (*N*)	LDL-C >130 mg/dL% (*N*)
This study	2019	3869	44.5 (1727)	47.8 (1853)	45.7 (1769)	41 (1589)
R. Gupta^,12^	2016	49904	25.4 (12676)	54.9 (27397)	33.9 (16917)	28.1 (1402)
Rajeev Gupta et al.^16^	2015	3426	24.8 (850)	34.1 (1168)	41.2 (1411)	16.3 (551)
T. Sekhri et al.^19^	2014	10642	32 (3405)	37.7 (741)	NA	NA
Women	Year	Sample size	TC >200 mg/dL%(N)	HDL-C <50 mg/dL % (*N*)	TG >150 mg/dL % (*N*)	LDL >130 mg/dL % (*N*)
This study	2019	579	31.9 (185)	65.8 (381)	22.1 (128)	29.4 (170)
R. Gupta^12^	2016	17491	35.6 (6227)	64.4 (11264)	26.8 (4688)	35.1 (6139)
Rajeev Gupta et al.^16^	2015	2772	25.3 (701)	53 (1469)	31.5 (873)	15.1 (413)
T. Sekhri et al.^19^	2014	1966	27.6 (543)	76 (1499)	NA	NA

(a) Comparison of mean lipid levels among Indian expatriates in Qatar with other studies on Indian residents in India. Total cholesterol, triglycerides, and LDL-C are higher in men in this study, while HDL-C is lower from studies in 2014 and lower from the 2016 study. (b) Comparison of prevalence of dyslipidemia in Indian expatriates with Indian residents in India. The prevalence of total cholesterol, triglycerides, and LDL-C dyslipidemia is high among Indian men in this study. Women expatriates showed lower prevalence from 2016 study and higher from 2015 to 2014.

## Data Availability

The data used to support the findings of this study are available from the corresponding author upon request.
